# Smartphone barometer can hear, and sense finger taps

**DOI:** 10.1186/s42400-025-00401-5

**Published:** 2026-01-21

**Authors:** Alireza Hafez, Dorsa Nahid, Majid Khabbazian

**Affiliations:** Department of Electrical and Computer Engineering, University of Alberta, Edmonton, Alberta Canada

**Keywords:** Smartphone, Barometer, Side-channel attack

## Abstract

Nearly all modern smartphones are now equipped with a barometer to sample air pressure. Accessing these samples is deemed harmless, hence does not require permission. In this work, we demonstrate that barometer samples can reveal sensitive information, particularly in smartphones with ingress protection. Using a support-vector machine (SVM) classifier, we demonstrate for the first time that barometer readings, even at a low sampling rate (25 Hz), can reveal smartphone speaker activity. In particular, our classifier achieves $\ge 95\%$ accuracy in detecting whether the speaker is silent or playing a ringtone. In addition, we show that low-rate barometer samples can be used to 1) detect touchscreen finger taps with nearly $100\%$ accuracy, and 2) gain information about the approximate position of finger taps. Our findings underscore that the barometer sensor, often considered harmless, should be recognized as sensitive with regard to user privacy, and access to this sensor should be carefully managed.

## Introduction

Smartphones have become an integral part of our daily lives. We have been increasingly relying on smartphones for various tasks including communications, navigation, fitness tracking, gaming, social networking and banking. To support this wide set of tasks, smartphones are equipped with an array of sensors ranging from microphones and cameras to GPS, accelerometers, gyroscopes, ambient-light, and barometers.

The abundance and sensitivity of data collected by sensors in smartphones is a major privacy concern. To address this concern, smartphones have put in place permission mechanisms that allow users to control apps’ access to many of these sensors. In Android, for example, apps must receive permission from users either at install time or at run time to access sensitive sensors. In addition, access to sensitive sensors may be taken away under certain conditions. For instance, an app in the background is not granted access to the touchscreen input, as the input may contain sensitive information such as passwords or texts that belong only to the foreground app.

While many sensors, such as microphones, require strict permissions, others are accessible without user approval or notification because their data is deemed safe. One such sensor is barometer, now standard in nearly all mid-range to high-range smartphones. Beyond measuring air pressure, barometers can be used as altimeters since pressure data can be translated to altitude. This can be used to improve (1) GPS accuracy in vertical direction (Zhang et al. 2012); (2) indoor navigation by revealing floor changes (Muralidharan et al. 2014); and (3) accuracy in apps such as fitness apps (Sankaran et al. 2014).

In addition to offering a barometer, nearly all mid-range to high-range smartphones now feature ingress protection, which guards against dust and liquid ingress. Smartphones with ingress protection are sealed and nearly air-tight. As a result, it takes time for a sudden change in the device’s internal air pressure to equalize with the air pressure of external environment. This potentially gives enough time to the smartphone’s barometer to capture a change in the internal atmosphere.

A sudden change in the internal atmosphere of a smartphone can happen as a result of, for example, pushing the device’s screen, or even playing a sound on the device’s earpiece speaker (after all, sound is a pressure wave). This raises the question of whether one can extract sensitive information, such as user’s finger tap (a gentle touch on the screen with a finger) and speaker’s activity, from the barometer samples.

A significant challenge in extracting such information is the low sampling rate of smartphone barometers. The Android Sensor API limits barometer sampling to 25Hz, even though hardware like the STMicro LPS22HH used in our experiments supports rates up to 200Hz (High-performance MEMS LPS22HH). Despite this constraint, we demonstrate, for the first time, two distinct side-channel information leaks in smartphones with ingress protection.

First, we show that barometer readings can reveal information about a smartphone’s speaker activity. Second, we demonstrate that barometer samples can detect finger taps with nearly 100% accuracy. Additionally, our findings indicate that barometer data leaks information about the position of finger taps.

These findings suggest that even at a limited sampling rate, barometer data can be exploited for sensitive information leakage. However, tasks such as estimating finger tap positions and detecting voice activity could become even more precise if access to higher sampling rates were available. Therefore, to mitigate the risk of more advanced side-channel attacks, restrictions on barometer sampling rates should either remain in place or be carefully regulated in the future.

This vulnerability allows a malicious app to continuously log barometer data in the background and analyze the samples to detect and locate finger taps. Since barometer access does not require user permission, the app could be disguised as an innocuous application, such as a weather or fitness app, and silently collect barometer data without raising suspicion.

Notably, our experiments show that excessive barometer access is not flagged as a potential threat by major antivirus software, including McAfee, Avast, and Norton. Without effective defense mechanisms, applications that rely on touchscreen input for sensitive data, such as PINs or credit card details, remain vulnerable to this side-channel attack. The risk is further increased when combined with other attacks, such as Simon and Anderson (2013), which uses the ambient light sensor to infer a user’s PIN input.

*Paper Organization.* The remainder of this paper is organized as follows. We review related work in Section "Related work", and present background information in Section " Background". We discuss our initial observations in Section "Initial observations", and present our experimental methodology and results in Section " Experimental Settings". We discuss an attack scenario in Section "A sample attack scenario", and explain security implications and defense solutions in Section "Security implications and mitigation". Finally, we conclude and present future research directions in Section " Conclusion and future work".

## Related work

Due to features such as the abundance of sensors in smartphones, side-channel attacks on these devices have received increasing attention (Spreitzer et al. 2017). Various smartphone sensors, including accelerometers (Aviv et al. 2012; Liu et al. 2015; Owusu et al. 2012), gyroscopes (Michalevsky et al. 2014; Wang et al. 2015), in-display fingerprint sensors (Ni et al. 2023) and microphones (Chen et al. 2020; Lu et al. 2019), have been leveraged to analyze associated side channels (see (Spreitzer et al. 2017) for a systematic classification of side-channel attacks on mobile devices).

Beyond sensors, smartphone auxiliaries have also been leveraged for side-channel attacks. For instance, Ni et al. (2023) utilized multi-port chargers to launch attacks on one port to eavesdrop or inject voice commands into other connected devices.

While side-channel information is often associated with security attacks, it has also been used to enhance security. For example, in Chen et al. (2022), the authors utilize the built-in audio modules of smartphones to improve the security of traditional pattern lock schemes. Similarly, Yao et al. (2023) employs barometer readings and ambient sensor data to develop a novel implicit authentication method

*Barometer.* Air pressure measurements by smartphones’ barometers can be used to estimate altitude, and detect altitude changes of as little as one meter (Sankaran et al. 2014). Because of this, barometers on smartphones have been used in many apps such as aiding GPS (Zhang et al. 2012), detecting floor level for indoor positioning (Vanini and Giordano 2013), and improving calorie estimation in fitness apps (Sankaran et al. 2014).

Smartphone barometers have not been explicitly used in the literature for side-channel attacks. Existing works, however, suggest that barometer’s measurements leak information about users’ activities and their surroundings. Although these works did not present their results as side-channel attacks, their use of the barometer sensor can be arguably considered as a side channel application. For instance, Sankaran et al. (2014) showed that barometer data can be used to detect user’s activities IDLE, WALKING, and in VEHICLE. In another work, Ho et al. (2015) exploited barometer data to infer driving routes. And, sudden changes in barometric data was utilized by Wu et al. (2015) to detect the buildings’ door opening/closing events.

Barometers have also been used to capture touchscreen pressure (Yao et al. 2023; Quinn 2019). In Yao et al. (2023), the authors used a digital dynamometer to measure force on a smartphone’s front panel and found a linear correlation between barometer readings and applied pressure. Leveraging this correlation, they designed an implicit authentication system that integrates barometric data with ambient sensors, and removed the need for system APIs in their approach to on-screen pressure estimation.

Similar to Yao et al. (2023); Quinn (2019) demonstrated that barometers in smartphones with ingress protection can estimate robotic forces on touchscreens. This finding led to a proposal to use barometers for touch force estimation to provide force sensing functionality in smartphones without dedicated force-sensing hardware. One of the contributions of our work is to show that barometers on such devices are sensitive enough to detect even gentle finger taps, and to some extent their positions.

*Voice recognition.* Motion sensors, such as the gyroscope and accelerometer, have been explored in the literature for speech recognition of an external speaker via solid or air vibrations (Michalevsky et al. 2014; Zhang et al. 2015). However, based on further analysis, the authors in Anand and Saxena (2018) argued that these threats may not extend beyond the loudspeaker setup examined in Michalevsky et al. (2014). Additionally, as demonstrated in Huang et al. (2024), attackers can exploit smartphone voice assistants for eavesdropping, a simpler and more potent threat than motion sensor-based attacks, which require additional equipment and complex signal processing.

In a different setup, the authors in Ba et al. (2020) leveraged the accelerometer as a side channel to capture speech signals played by the smartphone’s own speaker. They demonstrated that, in this setup, the attack is effective. We adopt the same setup as Ba et al. (2020); however, instead of a motion sensor, we utilize the barometer—an environmental sensor with a low sampling rate. To the best of our knowledge, this is the first study to explore the feasibility of detecting speaker activity, or sound in general, using a smartphone’s barometer.

## Background

### Ingress protection

The degree of ingress protection offered by a device is typically indicated by an IP (Ingress Protection) code from IEC standard 60529. An IP rate typically has two digits (Bloch 2009). The first digit refers to the ingress protection against solids. This scale goes from 0 to 6, where 0 indicates no protection, and 6 indicates dust-tight. The second digit indicates the protection level against liquid. The digit ranges from 0 to 9, where a higher number implies more protection. Most of the mid to high-end smartphones, including the smartphone used in our experiments, are either IP68 or IP67 certified. These ratings imply that they are dust-tight, and protected against immersion in liquid (Yu et al. 2019).

### Barometer and barometric vents

In devices with ingress protection, barometric vents are added to allow the internal barometer measure outside atmospheric pressure. The vents also protect the device’s seals and internal components from the stress that can be placed by the difference in air pressure inside and outside the device. The barometric vents are hydrophobic membrane, and are structured to prevent penetration of dust particle while allowing the flow of air to equalize internal and external barometric pressures (Yu et al. 2019). The air pressure equalization is, however, slow and occurs over a short period of time. This, as will be shown in this work, makes it possible to capture sudden changes to the internal pressure using low-rate barometric samples provided by the barometer.

**Fig. 1:**
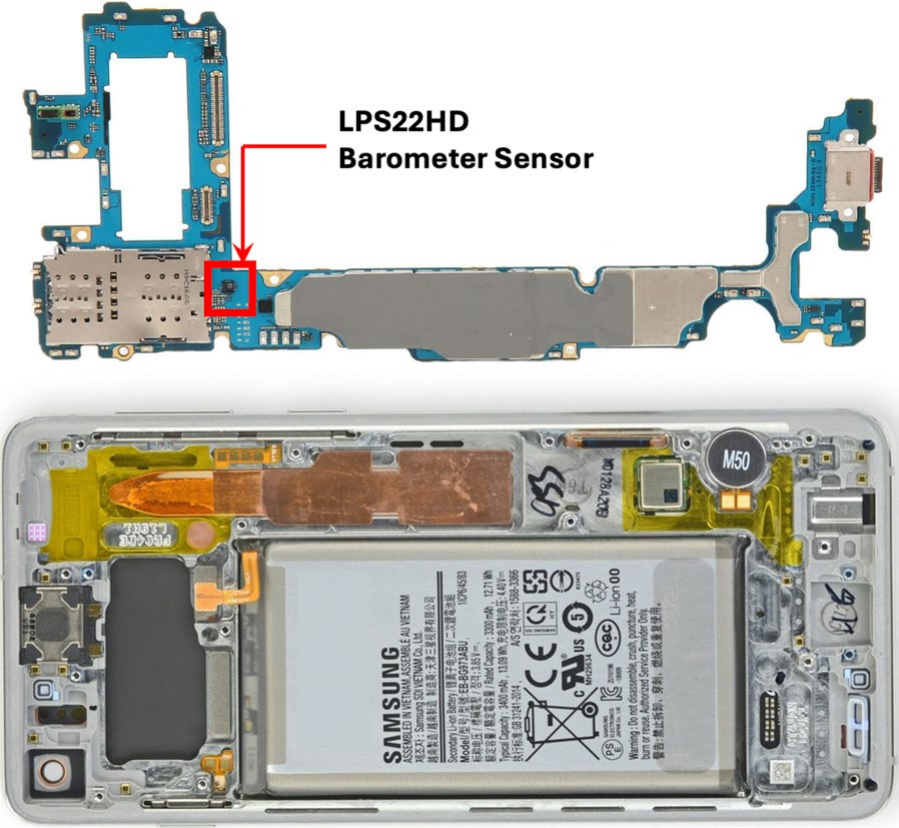
Samsung Galaxy S10 Plus barometer sensor (highlighted)

Fig. 1 shows the placement of the barometer sensor inside one of our testing smartphones, Samsung Galaxy S10 Plus. The barometer used in this smartphone is an ultra-compact LPS22HH pressure sensor by STMicroelectronics. According to the LPS22HH datasheet, the sensor is capable of sampling air pressure at 200 Hz (High-performance MEMS LPS22HH). However, the Android Application Programming Interface (API) limits the rate to 25 Hz (Sankaran et al. 2014). In this work, we access the barometer samples at the limited rate of 25 Hz.

### Earpiece speaker

**Fig. 2:**
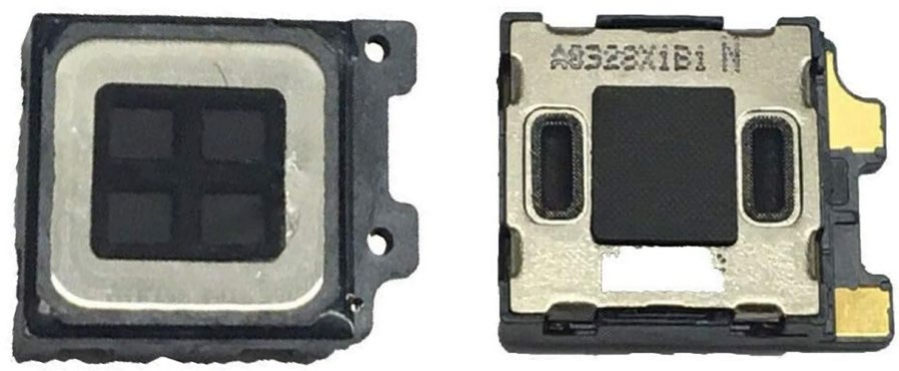
The Samsung Galaxy S10 Plus earpiece speaker with vent holes on both sides to allow air to move in and out

Fig. 2 shows the Samsung Galaxy S10 Plus speaker. Similar to earpiece speakers in other smartphones, vent holes on both sides of the speaker allow air to move in and out of the speaker when it plays a sound. Both the earpiece speaker and the barometer are inside the smartphone. Therefore, it is natural to ask if the barometer is able to capture the speaker’s activity, as sound is a pressure wave.

## Initial observations

As mentioned earlier, in devices with ingress protection, the internal pressure equalizes with the external pressure over a short period of time. This potentially opens a door to capture changes in the internal pressure using low-rate samples from the device’s barometer.

*Observation 1:* To have an initial verification of the above hypothesis, we played a 12 Hz sinusoid tone on the device’s earpiece speaker (we later replaced this tone with a ringtone). Note that 12Hz is slightly less than half of the the barometer’s 25Hz sample rate. Therefore, according to the Nyquist–Shannon sampling theorem, the discrete-time sequence obtained from the barometer’s samples will be free of aliasing. However, it will still be affected by noise, as barometric sensor data is susceptible to interference from various factors.

**Fig. 3:**
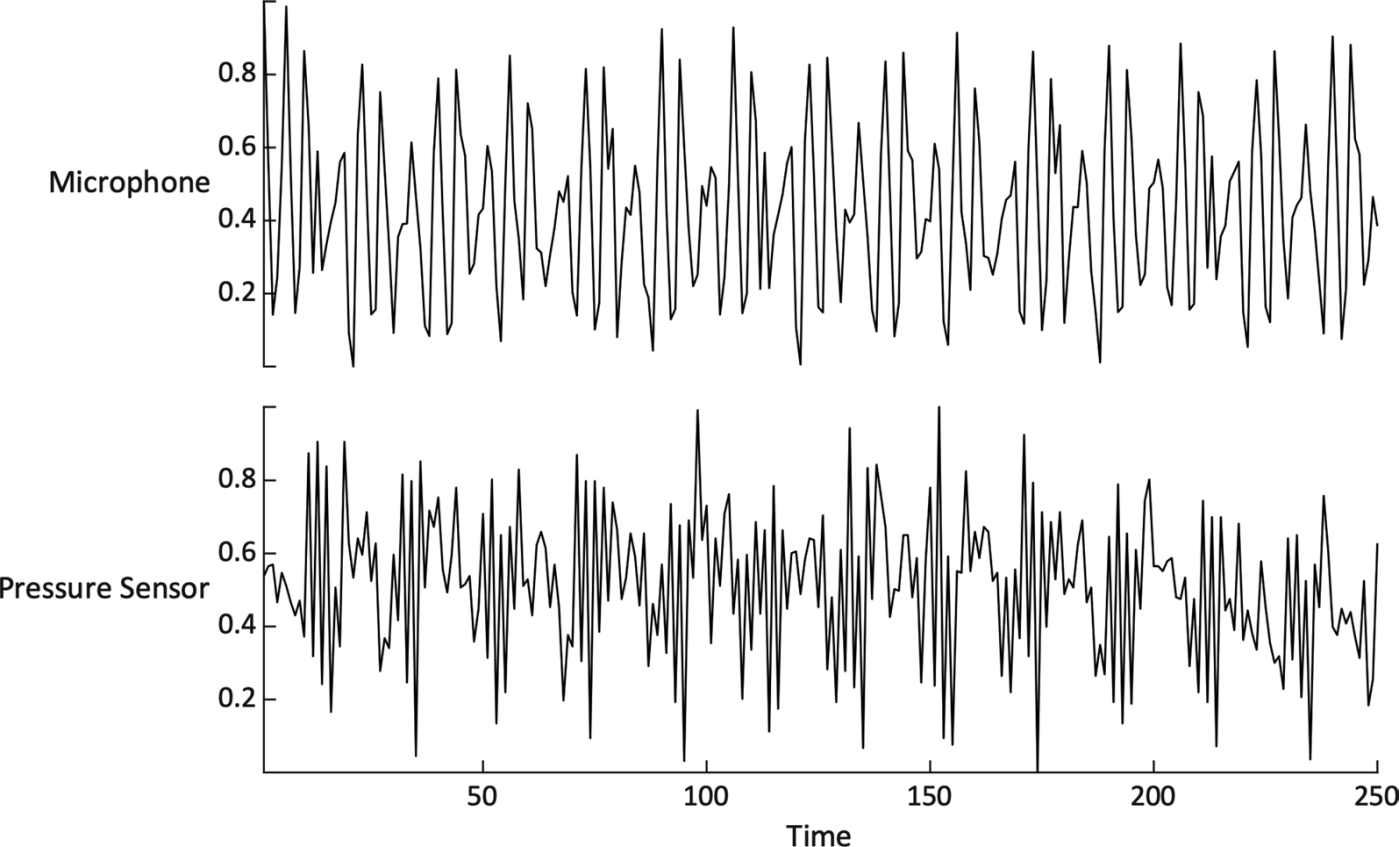
Normalized signals recorded by the device’s barometer and microphone while playing a 12 Hz sinusoidal tone through the earpiece speaker

Fig. 3 shows the barometer samples when a 12 Hz sinusoid tone was played on the device’s earpiece speaker. Notice that the barometer sample signal roughly follows the sinusoid signal recorded by the device’s microphone. This primary observation suggests that it may be possible to detect earpiece speaker activity using the device’s internal barometer.

**Fig. 4:**
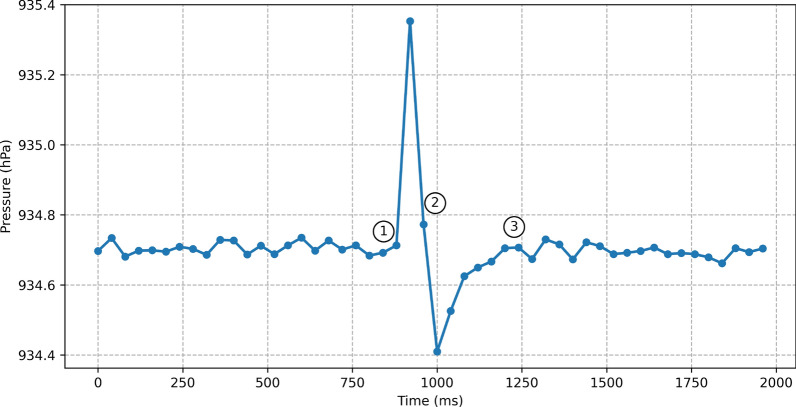
The impact of a finger tap on the internal pressure captured by the device’s barometer. (1) internal pressure increases as the result of finger tap, (2) a decrease in the pressure after the finger tap, (3) pressure equalization

*Observation 2:* A finger tap slightly flexes the screen inwards, causing the internal volume of the device to slightly decrease. Since the device is nearly airtight, the gas inside the device cannot escape immediately through the vents. Consequently, the gas inside the device is compressed, causing the internal barometric pressure to increase. Once the user removes the finger from the screen, the screen returns to its normal position and causes a short vacuum, this time causing the internal pressure to decrease. Fig. 4 shows the impact of a touchscreen finger tap on the internal pressure of our testing device. This initial observation suggests that low-rate barometer samples may leak information about finger taps.

**Fig. 5:**
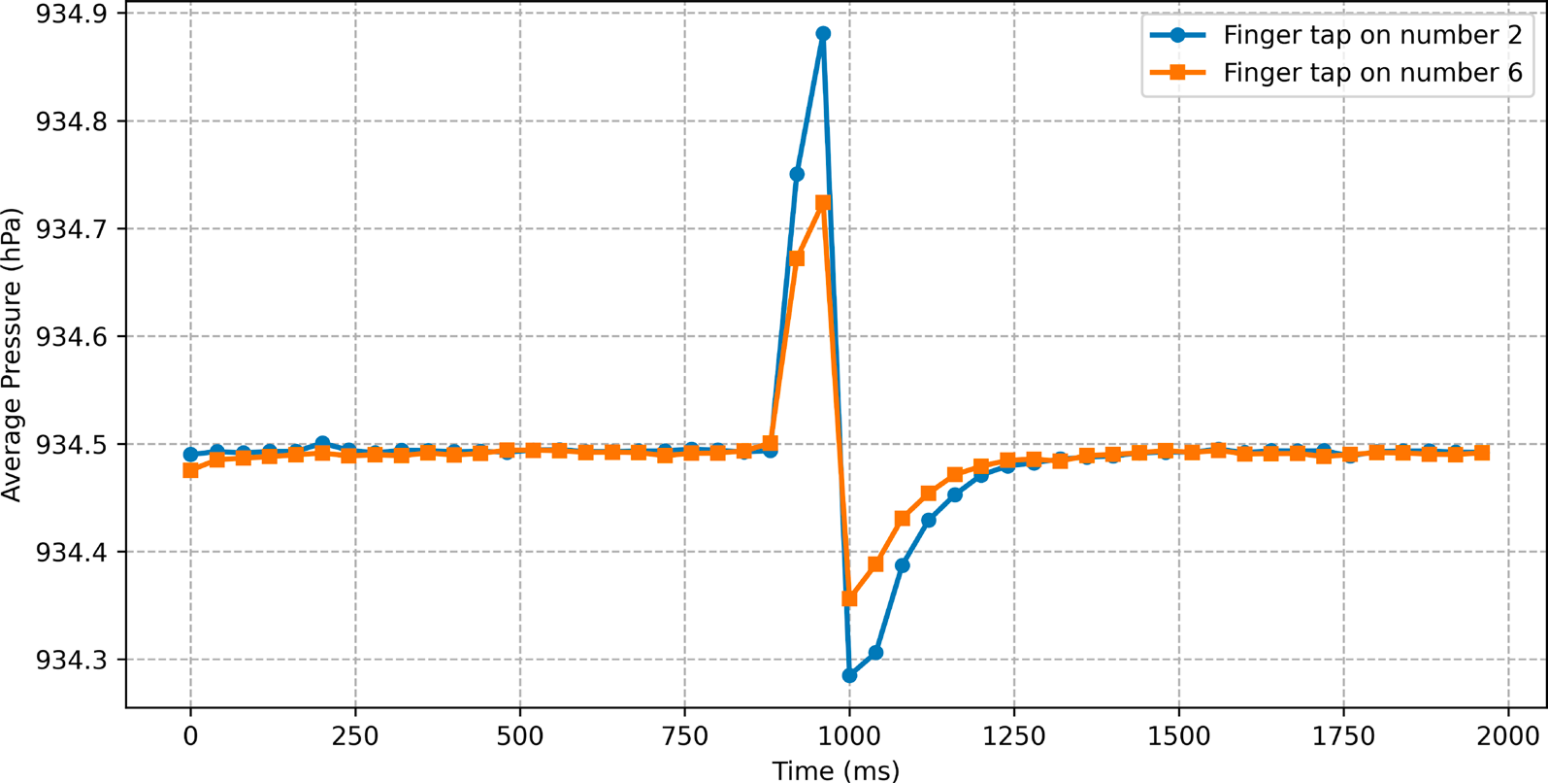
The average impact of a finger tap as the result of tapping on Number 2 and Number 6 of our custom numpad. The positions of these numbers are shown in Fig. 6

**Fig. 6:**
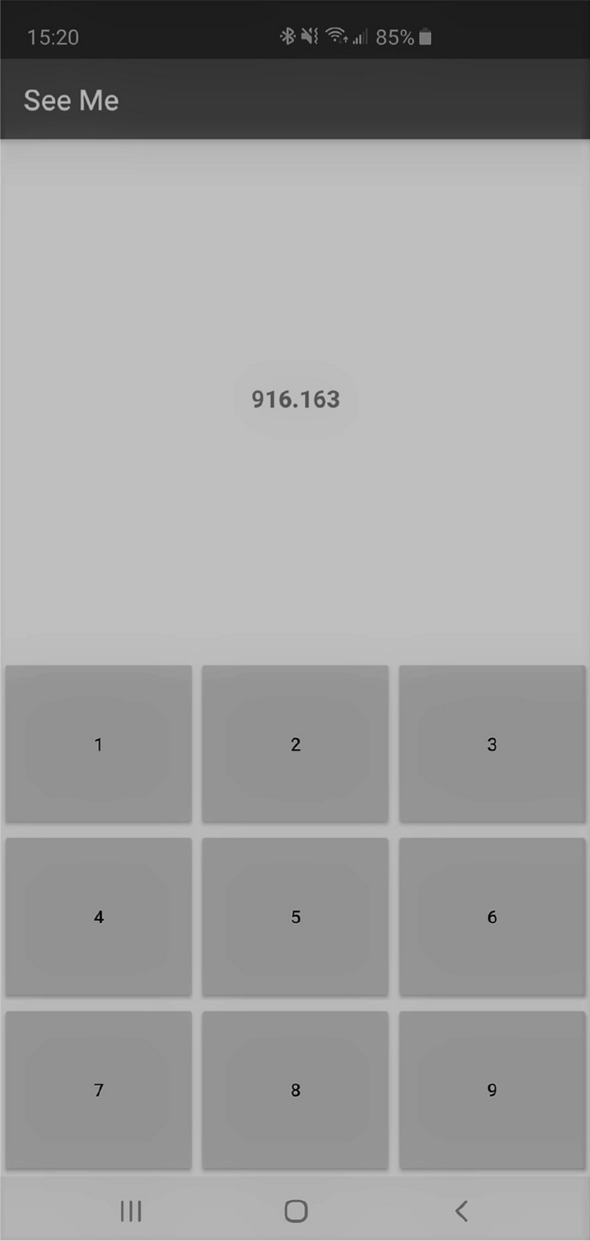
Pin entry keypad (numpad) in the TouchSpy app

*Observation 3:* Figure 5 show the average^1^ impact of a finger tap on two different positions on the touch screen (number 2 and number 6 in a custom numpad shown in Fig. 6). The variance for the peak and valley of the shown signals in the case of number 2 are 0.22 and 0.06, and for number 6 are 0.08 and 0.04, respectively. The difference between these two depicted impact signals suggests that it may be possible to detect the positions of finger taps on the touch screen. The main reason for the difference between these two impact signals is that taps on different positions of the touch screen can cause different amount of screen flex.

Considering the above initial observations, we aimed to answer the following questions:

1. Is it possible to detect any activity of earpiece speaker using the internal barometer of the same device in both IP and non-IP smartphones?
2. Is it possible to detect a gentle finger tap from the internal barometer samples?
3. Is it possible to gain information about the position of finger taps from the barometer samples?
4. Do common antivirus software detect excessive reading of the barometer pressure samples as a power virus?

We will answer these questions in the remaining of the paper.

## Experimental settings

### Device used

The presented results in this work were obtained from data collected on a Samsung Galaxy S10 Plus smartphone with ingress protection rating of IP68. Our primary choice of the Galaxy S10 was due to its ingress protection feature. However, it’s worth noting that any other Android device with ingress protection could serve a similar purpose. In fact, we observed comparable results from data obtained on Samsung Galaxy S21, Samsung Galaxy S21 Ultra, and Google Pixel 2 smartphones.

To address the second part of our first question, we used a Samsung Galaxy S6 Edge, which is a non-IP rated smartphone. The choice of the Galaxy S6 Edge was specifically because it lacks an IP rating. Any other non-IP rated Android smartphone could be used in this scenario.

We focused on Samsung and Google Pixel devices as these represent two major Android phone manufacturers with relatively consistent hardware across their product lines. This consistency allows for better control over potential hardware variations that could impact the effectiveness of our attacks (e.g., sensor placement, ingress protection rating). Additionally, this focus enabled a more in-depth analysis within the scope of the study.

### Data collection

We developed two custom Android apps for data collection: SpeakerSpy and TouchSpy. Using SpeakerSpy, we recorded barometric pressure samples during earpiece speaker activity (i.e., when the given sound is played) and inactivity (i.e., when the speaker is silent). During data collection, the test device was stationary on a desk. To collect data records, we alternated between ten seconds of speaker activity, and ten seconds of inactivity. A two-second “resting period” was added between consequent periods of speaker activity and inactivity to allow the internal and external air pressures to equalize. A data record was labeled *active* if the barometer samples were taken during the speaker activity; otherwise, it was labeled *inactive*. We collected a total of 450 records, half of which were labeled as active and the other half as inactive.

The second used app was TouchSpy. We used this app to read and record barometric pressure samples during a screen finger tap. Each record spans an interval of two seconds, starting one second prior to the tap. The barometer sampling rate is 25 Hz, thus each record consists of 50 samples. To collect real data using TouchSpy, we employed three participants. Each participant was asked to tap (as they normally do) the touchscreen of a stationary phone placed on a desk. In one setting, the participants were asked to tap a randomly selected position on the screen.

In the second setting, the aforementioned three participants were asked to tap a random number on a custom 3-by-3 pin entry keypad shown in Fig. 6. For each setting, we collected a total of 1116 records. The records collected in the first setting were used to train and test a classifier for detecting finger taps, while the ones collected in the second setting were used to train and test a classifier for inferring the position of a tap.

*Ethics.* As mentioned above, the data collected consists of finger taps on the touchscreen of a phone.

All participants were co-authors of this manuscript and provided informed consent to participate. The participants, all adults, were fully informed about the study, and verbal consent was obtained in the presence of at least two adult witnesses. No identifiable personal information was collected as part of this study. This research study has received ethics approval by the University of Alberta, Research Ethics Office, approval code Pro00146159.

### Data preprocessing

We treated each record as a time series, and normalized it to reduce/eliminate the impact of the measurement unit, signal power and gradual changes in atmospheric pressure in the course of data gathering. To this end, we used a simple normalization called standardizing, in which each data point *x* in the time series is replaced by its *z*-score:

$$\begin{aligned} z=\frac{x-\bar{x}}{S} \end{aligned}$$

where $\bar{x}$ and *S* are the mean and standard deviation of the time series, respectively.

### Classification and performance evaluation

We used a support-vector machine (SVM) as our classification method for the collected set of records. Each record is a time series composed of 50 samples, which are stored in an array of size 50. Each array corresponds to a feature, and the associated button press serves as the label for that feature. As described in Section "Data preprocessing", we normalize each feature prior to applying the SVM model. In our SVM implementation, we used a linear kernel and set the penalty parameter *C* to one.

To evaluate the accuracy of classification, we applied a 5-fold cross validation. In a 5-fold cross validation, the records are randomly split into five groups of equal size, where each group has equal number of records from each class. Then, for every number *i*, $1\le i \le 5$,

1. the group *i* is marked as the *test set*;
2. the remaining four groups are marked as the *training set*;
3. the SVM classifier is trained using the training set;
4. the accuracy of the trained classifier is measured using the test set.

The average of the five accuracy scores (obtained in Step 4) is returned as the accuracy result of the cross validation. In this work, all reported accuracy results are the average of running 5-fold cross-validation ten separate times. Each iteration of the cross-validation process was conducted independently, and the final accuracy results are averaged over these ten iterations. In addition to average accuracy, we report confidence intervals and minimum and maximum values.

## Results

### Earpiece speaker activity detection

Barometer-based speaker activity detection (B-SAD) can be stated as a binary classification problem: given a sound and the barometer samples, infer whether the smartphone’s speaker is playing the sound or is silent. Note that a malicious app with B-SAD capability can steal user’s sensitive information. For example, it can set the sound to, say, the device’s default ringtone to detect when a phone call is received.

To evaluate the accuracy of classification in solving B-SAD, we used two types of sounds. The first one was a simple sinusoid tone of 12 Hz (nearly half the barometer’s sampling rate of 25 Hz). For the second sound we used a popular Android ringtone. In our experiment, the SVM classifier achieved a detection accuracy of 96% for the 12 Hz sinusoid tone, and 95% for the default Samsung ringtone.^2^ We therefore infer that barometer samples can leak information about the smartphone’s earpiece speaker.

We further investigated whether similar results can be obtained on a non-IP device.

To show this, we repeated the above experiment with Samsung Galaxy S6 Edge, a non-IP rated smartphone. In this experiment, our SVM classifier achieved a detection accuracy of 67%. This accuracy is non-negligible, although it is significantly lower than the 95% accuracy we achieved in the IP68-rated smartphone. Based on the above results, barometer samples in non-IP smartphones, too, can leak information about the earpiece speaker activity.

### Detecting finger taps

Unlike the five-second robotic arm touch contacts in Quinn (2019), finger taps have a short touchscreen contact time. In our experiment, the touchscreen contact time of a finger tap ranged from 24 ms to 183 ms, with the average of 85 ms. As stated in Quinn (2019) if the input force changes too quickly, it may not be captured by the barometer. In addition, based on the study in Taher et al. (2014), finger taps typically produce less force than those applied in Quinn’s experiment. Therefore, it is not clear if finger taps are detectable at all.

To study whether finger taps are detectable, we conducted an SVM classification on the data records collected by our TouchSpy app. To evaluate the accuracy of classification, as before, we applied multiple 5-fold cross validations. Despite the short contact time and low force level of finger taps, our classifier achieved full detection, i.e. detected finger taps with 100% accuracy.

### Detecting the position of a tap

In the previous section, we showed that barometer samples can accurately detect a finger tap on the touchscreen. This brings us to the next question of whether or not barometer samples leak information about the position of finger taps.

Our initial observations (Observation 3 in Section 4) suggest that barometer samples may leak information about the position of finger taps. To verify this hypothesis, we used a custom numpad to collect finger tap records as reported in Section 5.2.

The designed numpad (Fig. 6) uses exclusively the lower half of the screen, as this is the default location of touch keyboards and numpads on most smartphones. The numpad divides the lower half of the screen into nine different regions/keys. Each region/key has different degrees of freedom. The difference in degrees of freedom leads to different amounts of screen flex and recovery as the result of a tap. This makes it possible to profile each region/key, and guess the number which was tapped.

The number in row *i* and column *j* of the table is the average of the percentage relative frequency that a classifier outputs *i* when the actual number tapped was *j*. For instance, by Table 1, when user presses 5 our SVM classifiers, on average, output 2, 3, 4, 5, and 6 with relative frequencies of 17.36%, 14.92%, 6.69%, 38.55% and 22.33%, respectively. The numbers on the main diagonal of Table 1 represent the empirical probabilities of correct identification. These probabilities vary from $27.73\%$ to $48.46\%$. By averaging these diagonal entries, we get that if a digit is chosen uniformly at random from the numpad, the SVM classifier correctly identifies it with a probability of $36.44\%$. This accuracy significantly exceeds the probability of correctly guessing a random number, which is $\frac{1}{9} \approx 11\%$.

The performance of a classifier can be summarized in a table, such as Table 1, which we will refer to as the performance table of the classifier. Tables 2, 3, 4 and 5 represent, respectively, the minimum, maximum, lower, and upper confidence intervals aggregated across the performance tables of all classifiers in our simulations.

To view the significance of this side-channel information leakage from a different perspective, we can estimate the mutual information between the random variable *X* representing the number tapped on the numpad, and the random variable *Y* representing the output of our SVM classifier.

Suppose *X* has uniform distribution. Taking the frequency distribution presented in Table 1 as the conditional probability distribution of *Y* given *X*, we can estimate the mutual information between *X* and *Y* as

$$\begin{aligned} \begin{aligned} I(X;Y)&= H(Y)-H(Y|X)\\&\approx 3.16-2.15\\&= 1.01 \text { bits}, \end{aligned} \end{aligned}$$

where *I*(*X*; *Y*) denotes the mutual information between *X* and *Y*, and *H* is the entropy function. Therefore, when the random variable *X* follows a uniform distribution, observing the random variable *Y* (i.e., the output of the classifier) leaks approximately one bit of information about *X*, which has an entropy of $H(X) = \log _2 9 \approx 3.17$ bits.

**Table 1:** The number in row *i* and column *j* of the table is the average percentage of relative frequency that the classifier returns *i* when the actual number tapped was *j*

	#1	#2	#3	#4	#5	#6	#7	#8	#9
#1	27.73	0.08	0.00	6.85	0.08	1.29	20.31	16.07	21.97
#2	0.00	45.23	28.37	0.00	17.36	2.50	0.00	0.00	0.00
#3	0.00	20.16	36.58	0.08	14.92	5.33	0.00	0.00	0.00
#4	7.16	1.77	1.85	43.25	6.69	15.82	8.13	7.82	4.92
#5	0.00	20.48	17.65	0.97	38.55	22.41	0.00	0.00	0.00
#6	0.00	10.66	14.16	19.52	22.33	48.46	0.00	0.00	0.00
#7	22.60	0.73	0.41	16.27	0.00	1.29	27.82	27.29	19.96
#8	15.35	0.80	0.97	10.56	0.08	2.57	25.61	30.79	23.61
#9	27.16	0.08	0.00	2.50	0.00	0.32	18.13	18.04	29.55

**Table 2:** The number in row *i* and column *j* of the table is the min percentage of relative frequency among the classifiers

	#1	#2	#3	#4	#5	#6	#7	#8	#9
#1	23.37	0.00	0.00	5.60	0.00	0.08	16.10	14.50	19.40
#2	0.00	43.50	24.10	0.00	13.73	1.60	0.00	0.00	0.00
#3	0.00	17.80	33.03	0.00	13.73	4.00	0.00	0.00	0.00
#4	5.63	0.80	0.80	41.13	5.60	13.63	6.43	6.40	3.27
#5	0.00	17.67	12.83	18.47	20.13	44.30	0.00	0.00	0.00
#6	0.00	8.90	12.83	18.47	20.13	44.30	0.00	0.00	0.00
#7	18.60	0.00	0.00	14.50	0.00	0.80	24.93	21.80	14.47
#8	10.47	0.00	0.80	8.80	0.00	1.60	17.90	27.40	20.10
#9	19.33	0.00	0.00	0.80	0.00	0.00	15.27	14.50	25.07

**Table 3:** The number in row *i* and column *j* of the table is the max percentage of relative frequency among the classifiers

	#1	#2	#3	#4	#5	#6	#7	#8	#9
#1	32.97	0.80	0.00	8.03	0.80	1.63	24.17	18.50	24.30
#2	0.00	48.47	33.77	0.00	19.53	3.23	0.00	0.00	0.00
#3	0.00	21.87	38.77	0.80	15.40	8.10	0.00	0.00	0.00
#4	8.83	3.20	2.47	45.13	7.33	17.80	10.50	9.60	5.67
#5	0.00	22.57	22.63	1.63	41.93	24.93	0.00	0.00	0.00
#6	0.00	12.93	15.30	21.00	25.03	51.67	0.00	0.00	0.00
#7	25.77	0.83	0.83	19.40	0.00	1.63	32.20	32.30	24.10
#8	20.20	1.63	1.67	12.90	0.80	4.03	29.07	34.67	29.03
#9	33.07	0.80	0.00	4.07	0.00	0.80	20.90	22.57	33.87

**Table 4:** The number in row *i* and column *j* of the table is the lower bound of 95 percent confidence interval of relative frequency

	#1	#2	#3	#4	#5	#6	#7	#8	#9
#1	25.35	$-$ 0.10	0.00	6.23	$-$ 0.10	0.99	18.45	14.94	20.86
#2	0.00	44.22	26.41	0.00	16.15	2.07	0.00	0.00	0.00
#3	0.00	19.20	35.33	$-$ 0.10	14.52	4.37	7.17	7.02	4.42
#4	6.43	1.32	1.46	42.27	6.21	14.76	7.17	7.02	4.42
#5	0.00	19.44	15.74	0.51	37.00	21.21	0.00	0.00	0.00
#6	0.00	9.81	13.55	18.81	21.07	46.75	0.00	0.00	0.00
#7	20.65	0.55	0.10	15.06	0.00	0.99	26.13	25.02	17.41
#8	13.06	0.53	0.72	9.55	$-$ 0.10	1.92	23.35	28.91	21.27
#9	24.44	$-$ 0.10	0.00	1.75	0.00	0.02	16.73	16.49	27.33

**Table 5:** The number in row *i* and column *j* of the table is the upper bound of 95 percent confidence interval of relative frequency

	#1	#2	#3	#4	#5	#6	#7	#8	#9
#1	30.11	0.26	0.00	7.47	0.26	1.59	22.18	17.20	23.07
#2	0.00	46.25	30.34	0.00	18.57	2.93	0.00	0.00	0.00
#3	0.00	21.12	37.83	0.26	15.32	6.29	0.00	0.00	0.00
#4	7.90	2.23	2.25	44.22	7.16	16.89	9.09	8.62	5.42
#5	0.00	21.51	19.56	1.43	40.10	23.60	0.00	0.00	0.00
#6	0.00	11.51	14.78	20.23	23.58	50.17	0.00	0.00	0.00
#7	24.54	0.92	0.71	17.48	0.00	1.59	29.51	29.56	22.51
#8	17.65	1.08	1.22	11.56	0.26	3.22	27.66	32.67	25.94
#9	29.87	0.26	0.00	3.25	0.00	0.62	19.53	19.59	31.77

*Antivirus detection.* Barometer sensor’s power usage is normally low. Therefore, excessive reading of the barometer pressure samples may not be detected by common antivirus software as, for example, a power virus. To confirm this, we ran a background service that continuously reads barometric pressure samples for over 40 h. This service was not detected as a suspicious activity by McAfee, Avast, AVG, Bitdefender and Norton antivirus.

## A sample attack scenario

Traditional side-channel attacks, such as those in Van Eck (1985), typically require the adversary to have physical access to the device or be in close proximity to it. However, with the advent of smartphones, the attack landscape has shifted.

An adversary can launch an attack using a spy app that collects barometer samples in the background and analyzes the collected samples to extract sensitive information. As explained in Ba et al. (2020), the spy app can be disguised as any type of mobile application since accessing the barometer does not require any permission.

In more detail, the following steps can be taken to execute a successful touchscreen tap inference or B-SAD attack on a smartphone.

1. *Embed a malicious code into an app *. An adversary has at least two options to access the barometer’s API. The first option is to develop a new app (e.g., a weather forecast app) from scratch, and embed the malicious code (to gather barometer samples, and perform inference) inside it. The second option is to inject the malicious code into a popular and well-known app such as a popular game. There are pros and cons to each method. Developing an app from scratch gives the attacker the ability to publish the malicious app silently via, say, the Google Play Store. Also, it gives the attacker the chance to request user permission (such as Internet access) that may initially look legitimate. Such accesses can be beneficial. For example, the Internet access can be used to transfer the inferred user’s information to the attacker. By injecting the malicious code into a well-known and popular app, on the other hand, the attacker may hit a larger population. This option, however, does not allow the attacker to put the modified app on a trusted source like Google Play Store. This makes it harder for the app to find its way to the users’ phones.
2. *Perform data gathering and training*. Data gathering does not require any permissions from the user, but needs the user to interact with the malicious app. With an invisible overlay layout on top of the malicious app, the app can gather the necessary data for training a model for the user’s typing habits (to extract the exact force of user’s finger taps, for example) and phone screen size. In our experiments, about 50 samples per region (key) was the minimum for our classification to be viable. As for B-SAD, embedded sound queues in the malicious app are enough to train the model. In our experiments, to perform an accurate B-SAD, a minimum of 25 samples of each state (active/silence) was necessary. An adversary can use the gathered data to train an SVM classifier either locally at the victims’ device, or remotely at their own end.
3. *Perform inference and extraction*. At this point, the malicious app continuously logs the barometer samples in the background. At the same time, it feeds the collected samples to its classifier to detect taps and/or earpiece speaker activity. The results can be stored locally, or sent to the adversary over the Internet.

### Limitations

Our results confirm the possibility of detecting earpiece speaker activity and touchscreen taps using low-rate barometric air pressure samples. These results rely on relative pressure rather than absolute pressure values of the barometer; thus, they are applicable to different locations/altitude levels, and weather conditions. However, there are certain limitations in detecting speaker activity and finger taps using low-rate barometer samples. First, accuracy of detection depends on whether the device has ingress protection. It is because, otherwise, changes in internal pressure (due to, for example, finger taps) would quickly equalize the external atmosphere.

The second limitation is due to the low sampling rate of the barometer sensor. The STMicro LPS22HH sensor datasheet shows that the sensor is capable of sampling at 200 Hz. The Android sensor API, however, limits the barometer sample rate to 25 Hz^3^ Sankaran et al. (2014). In our experiments, the low sampling rate of 25 Hz was not a limiting factor in detecting earpiece speaker activity and detecting finger touch contacts. There are, however, certain tasks that are impossible to perform at low sampling rates. For instance, by the Nyquist–Shannon sampling theorem, it is impossible to reconstruct the speaker’s sound signal fully if the signal has frequencies higher than half the sampling rate of the barometer.

The low sampling rate of barometer can be preventive in carrying certain tasks related to screen touch detection as well. For example, in our experiments, we needed at least three sample points in our signal to register a finger tap, and then wait for at least two sampling cycles for the internal and external air pressures to equalize. At the sampling rate of 25 Hz, these five sampling cycles translate to a total of 200 ms, which limit the continuous detection of finger taps to five taps per second.

Finally, the flow rate of the barometric vent can also become a limiting factor. As stated in Quinn (2019), if a user’s input force changes either too quickly or too slowly, it may not be captured by the barometer.

## Security implications and mitigation

Modern mobile platforms such as Android allow apps to access the device’s barometer sensor without user’s permission or notification. Consequently, a malicious app can readily bypass the user’s permission and attention. Moreover, as shown in Simon and Anderson (2013), sensor-based side-channel attacks can bypass strong separation mechanisms like Samsung KNOX, which try to provide a secure environment to protect corporate data on smartphones.

In addition, due to barometer’s low power usage, accessing the barometric pressure samples may not be detected as, for example, a power virus. We ran a background service that continuously reads barometric pressure samples for over 40 h. This service was not detected as a suspicious activity by McAfee, Avast, AVG, Bitdefender and Norton antivirus.

*Mitigation strategies.* A first line of defense is to use the device’s permission system to ask users for permission to access the device’s barometer. This gives users the chance to deny permission to apps whose motivation behind requesting the access is not clear. For instance, users can deny barometer access to a “flashlight app” as such access is clearly outside the scope of the app’s functionality.

Another defense is to use third party apps such as AppGuard (Backes et al. 2013) that enforce security policies. These apps can pause sensor readings (or even kill) background processes/services when an app with sensitive input (e.g. a banking app) is running. In addition, malware-analysis apps such as Felt et al. (2011) and Gibler et al. (2012) can be extended and used to check for malicious sensor accesses.

Finally, sensitive apps can implement their own custom security solution. For example, a PIN pad can rearrange its buttons prior to every sensitive input (Owusu et al. 2012).

## Conclusion and future work

Speaker and touchscreen inputs can contain sensitive information. Because of this, touchscreen input is only given to foreground apps, and microphone access (which reveals speaker activity) requires user permission. In contrast, any app can access barometer samples without any permission or notification. This is alarming, as our results indicate that low-rate barometric pressure samples can reveal information about earpiece speaker activity. Additionally, the pressure samples leak information about finger taps and their positions.

Furthermore, as we reported in this work, common antivirus software does not flag excessive reading of the barometer sensor as suspicious activity. Consequently, a malicious app running in the background can gain sensitive information from the foreground app by monitoring and analyzing barometer sample data without being detected. Therefore, contrary to the existing belief that barometer’s samples are harmless, our findings underscore that the barometer sensor should be considered sensitive, and access to this sensor should be carefully managed.

*Future work.* Our work is a first feasibility study, and there are many ways to extend and improve our results. First, we only used SVM as our classifying method. More advanced classifiers and techniques can improve our reported accuracy results.

In detecting finger taps and their positions, we used a relatively small number of participants to collect data. Future research should include a larger number of participants to more comprehensively assess and validate the experimental findings. Also, in our experiments, we used a limited number of smartphones including Samsung Galaxy S10 Plus, Samsung Galexy S21, Samsung Galexy S21 Ultra and Google Pixel 2. We anticipate similar results in other smartphones with ingress protection. However, further studies and experiments are necessary to confirm this and enhance the generalizability of the findings by examining a wider range of hardware and sensor configurations across different brands.

Our results demonstrate high accuracy in detecting finger taps and sounds using low-rate barometer samples. However, when determining the precise position of finger taps, our accuracy is approximately 36%. While this represents a non-negligible information leak—more than three times better than random guessing—there remains significant room for improvement. One potential enhancement could involve integrating additional sensors, such as ambient light sensors that do not require access permissions, with the barometer.

With regards to earpiece speaker activity, it is interesting to see how many different classes of activities we can distinguish using low-rate barometric pressure samples. Our initial observations, not reported in this work, indicates that it may be possible to distinguish different sounds. Finally, it is interesting to see if it is possible to detect external sounds from silence using barometer’s low-rate samples.

## Data Availability

The datasets supporting the conclusions of this article are included within the article and its additional files.

## Footnotes

1 Averaged over 50 samples for each point on the *x*-axis.

2 Over-the-horizon ringtone, extracted from Samsung Galaxy S10 Plus.

3 The sampling rate of 25 Hz is provided by the SensorManager.SENSOR DELAY FASTEST in Android SDK.
